# Structural Maintenance of Chromosomes 5/6 Complex Is Necessary for Tetraploid Genome Stability in *Arabidopsis thaliana*

**DOI:** 10.3389/fpls.2021.748252

**Published:** 2021-10-05

**Authors:** Fen Yang, Nadia Fernández Jiménez, Joanna Majka, Mónica Pradillo, Ales Pecinka

**Affiliations:** ^1^Institute of Experimental Botany, Czech Academy of Sciences, Centre of the Region Haná for Biotechnological and Agricultural Research, Olomouc, Czechia; ^2^Department of Cell Biology and Genetics, Faculty of Natural Sciences, Palacký University, Olomouc, Czechia; ^3^Department of Genetics, Physiology and Microbiology, Faculty of Biology, Universidad Complutense de Madrid, Madrid, Spain; ^4^Institute of Plant Genetics, Polish Academy of Sciences, Poznań, Poland

**Keywords:** SMC5/6 complex, polyploidy, seed development, meiosis, *NSE2*, genome stability

## Abstract

Polyploidization is a common phenomenon in the evolution of flowering plants. However, only a few genes controlling polyploid genome stability, fitness, and reproductive success are known. Here, we studied the effects of loss-of-function mutations in NSE2 and NSE4A subunits of the Structural Maintenance of Chromosomes 5/6 (SMC5/6) complex in autotetraploid *Arabidopsis thaliana* plants. The diploid *nse2* and *nse4a* plants show partially reduced fertility and produce about 10% triploid offspring with two paternal and one maternal genome copies. In contrast, the autotetraploid *nse2* and *nse4a* plants were almost sterile and produced hexaploid and aneuploid progeny with the extra genome copies or chromosomes coming from both parents. In addition, tetraploid mutants had more severe meiotic defects, possibly due to the presence of four homologous chromosomes instead of two. Overall, our study suggests that the SMC5/6 complex is an important player in the maintenance of tetraploid genome stability and that autotetraploid Arabidopsis plants have a generally higher frequency of but also higher tolerance for aneuploidy compared to diploids.

## Introduction

Maintenance of genome stability is essential for ensuring plant growth, fertility, and proper genomic constitution of the offspring (Roy, 2014; Hu et al., 2016). The family of Structural Maintenance of Chromosomes (SMC) complexes includes ATP-dependent molecular machines with a unique ability to process chromosome-scale DNA molecules (Uhlmann, 2016). The SMC5/6 complex is an evolutionarily conserved member of the SMC family that is involved in DNA damage repair, DNA replication, and cell divisions (Kegel and Sjögren, 2010; Aragón, 2018). The core part of the complex consists of SMC5 and SMC6 protein heterodimer, where the subunits are attached via their hinge domains. SMC6 has two partially functionally redundant paralogs in Arabidopsis. Both play roles under ambient conditions, but only SMC6B takes place in DNA damage repair (Watanabe et al., 2009; Yan et al., 2013; Zou et al., 2021). Opposite to the hinge domains are the head domains, where the NON-SMC ELEMENT (NSE) NSE1-NSE3-NSE4 sub-complex bridges the SMC heterodimer. Here, the kleisin type protein NSE4 closes the SMC ring by interacting with both SMC5 and SMC6 subunits (Palecek and Gruber, 2015). The NSE1 and NSE3 subunits regulate the conformation of NSE4. While *NSE1* and *NSE3* are single-copy genes in Arabidopsis, there are two NSE4 paralogs (NSE4A and NSE4B) that show distinct expression patterns and functions (Díaz et al., 2019). The NSE2 subunit is attached to the coiled-coil region of SMC5 and is one of the two E3 SUMO ligases in Arabidopsis (Ishida et al., 2012). Additionally, plant-specific SMC5/6 subunits ARABIDOPSIS SNI1 ASSOCIATED PROTEIN 1 (ASAP1) and SUPPRESSOR OF NPR1-1; INDUCIBLE 1 (SNI1) have been described (Yan et al., 2013). ASAP1 and SNI1 were proposed to be functionally homologous to the yeast SMC5/6 complex chromatin-loader subunits NSE5 and NSE6.

SMC5/6 complex controls multiple biological processes in plants. There is solid evidence that the SMC5/6 complex is important for the repair of specific types of DNA damage in Arabidopsis (Mengiste et al., 1999; Watanabe et al., 2009; Yuan et al., 2014; Díaz et al., 2019). This may be mainly due to its essential role in homologous recombination (HR) where the loss of function from SMC6B results in reduced HR levels (Mengiste et al., 1999; Watanabe et al., 2009). NSE2 function is not essential for Arabidopsis survival, but the plants are strongly affected in their vegetative and generative development including poor growth of roots, earlier flowering, reduced height, and decreased fertility (Huang et al., 2009; Ishida et al., 2009; Xu et al., 2013; Liu et al., 2014; Kwak et al., 2016). Recently, several studies pointed toward the importance of the SMC5/6 complex during plant sexual reproduction, including meiosis, pollen viability, and seed development (Liu et al., 2014; Díaz et al., 2019; Yang et al., 2021; Zou et al., 2021). NSE2, NSE4, and SNI1 were found to play an important role in meiosis. NSE4A was localized to the synaptonemal complex and the mutants showed chromosome fragmentation and frequent meiotic irregularities (Zelkowski et al., 2019). At least part of this trait seems due to the role of SMC5/6 in the regulation of meiotic recombination. Here, RAD51 directly suppresses the SMC5/6 complex to promote DMC1-based recombination (Chen et al., 2021). Another role of the SMC5/6 complex is to secure the development of properly reduced haploid gametes in meiotic recombination independent manner (Yang et al., 2021). The NSE2, NSE4A, and SNI1 mutants show recombination-independent problems in chromosome segregation and produce unreduced microspores. Fertilization with diploid pollen leads to abnormal seed development in these mutants. This is most likely due to an unbalanced parental dosage with two maternal and two paternal genome copies in the endosperm (Jullien and Berger, 2010). An excess of paternal genetic information leads to seed overgrowth and the absence of cellularization, which frequently results in seed abortion (Köhler et al., 2012). Some of such abnormal seeds still survive and produce polyploid (triploid) offspring.

Polyploidization, i.e., whole genome duplication, is a common phenomenon in higher plants, and both autopolyploids and allopolyploids often occur in nature. Polyploidization plays a significant role in the evolution of Angiosperms as the major mechanism providing raw material for gene sub- and neofunctionalization (Van De Peer et al., 2009). However, newly established polyploids can experience genomic shock represented by changes at genomic, chromosome, and gene levels (reviewed in Comai, 2005). This includes genome down-sizing, structural chromosome rearrangements, amplification and/or reactivation of repetitive elements, modifications of the gene expression patterns, and rapid sequence changes in multigene families, such as rDNAs. Polyploidization often leads to altered morphology compared to the ancestral lines and in autotetraploid occasionally also to developmental abnormalities and/or reduced fertility. One of the major challenges in tetraploids is thought to be the more complex meiosis due to the presence of the four nearly identical (homologous chromosomes, autopolyploids) or similar (homeologous chromosomes, allopolyploids) copies of chromosomes. In both types of polyploids, natural selection should favor strategies to control pairing preferences that result in disomic inheritance and proper segregation of genetic material during meiosis. This is true in Arabidopsis, where auto- and allotetraploids show strict homologous chromosome pairing (Pecinka et al., 2011). The elimination of certain sequences, chromosome rearrangements, and dysploidy seem to contribute to the meiotic cytological diploidization (Mandáková and Lysak, 2018). Since it is a long process, intermediate situations with different chromosomes showing different rates of bivalent formation (tetrasomic inheritance for some chromosomes and disomic inheritance for others) are possible (Santos et al., 2003). Recently, it has been found that particular alleles of the meiotic chromosome pairing genes *ASY1* and *ASY3* lead to a reduced number of quadrivalents compared to bivalents in tetraploid *Arabidopsis arenosa* (Morgan et al., 2020; Seear et al., 2020), indicating an evolutionary selection toward specific tetraploid meiotic phenotypes.

Despite these findings, it remains largely unknown whether tetraploid mutants of meiotic genes show diploid-like or new phenotypes. This may contribute to a better understanding of their role in meiosis. Here, we analyzed the consequences of polyploidy in the SMC5/6 complex mutants and show that autotetraploid plants of two SMC5/6 complex mutants *nse2* and *nse4a* display several characteristics that differ from their diploid cytotypes.

## Materials and Methods

### Plant Materials and Growth Conditions

All strains used in this study were in Columbia-0 (Col-0) background. We used following mutants (diploid): *nse2-1/hpy2-1*, *nse4a-2* (GK-768H08), and *qrt1-4* (SALK_024104C). *nse2-1* is an ethyl methanesulfonate (EMS) mutant allele that was isolated in the laboratory of Prof. Keiko Sugimoto, RIKEN Center for Sustainable Resource Science, Japan. Other T-DNA insertion mutants were collected from the Salk Institute Genomic Analysis Laboratory (SiGnAL^1^; Alonso et al., 2003), and provided by the Nottingham Arabidopsis Stock Centre (NASC). Genotyping of T-DNA mutant was performed by PCR with a combination of three primers, T-DNA specific primers: LBb1.3 (5′-ATTTTGCCGATTTCGGAAC-3′) for *qrt1-4*; o84747_m (5′-ATAATAACGCTGCGGACATCTAC-3′) for *nse4a-2*, and two specific primers for the corresponding gene: LPNSE4A-2 (5′-GCTCAACAGGCGGTCATTTG-3′) and RPNSE4A-2 (5′-ACAAAAGCCACTTAACTGCTACA-3′); LPQRT1-4 (5′-TCTCTTCCCAGAAAAGGCTTC-3′) and RPQ RT1-4 (5′-CGTGGGTCTCAAGAATCTTTG-3′); *nse2-1* plants were selected based on the mutant features (Ishida et al., 2009). Double mutants were generated by crossing and selection in F_2_ and F_3_ generations. All lines were used as homozygotes unless stated otherwise.

Data related to diploid controls were published in a separate study (Yang et al., 2021). Both diploid and tetraploid plants were cultivated under the same growth conditions. Tetraploid *A. thaliana* plants were generated by submerging 2 weeks old *in vitro* grown diploid plants in 0.1% (w/v) colchicine (Sigma-Aldrich) in dark at room temperature for 1 h. Subsequently, plants were gently washed with copious amounts of tap water, transplanted to soil, and grown until maturity. Seeds were collected from individual plants, 20–30 biggest seeds were manually selected and propagated into plants for ploidy measurements (see below).

For *in vitro* growth, Arabidopsis seeds were surface sterilized (70% ethanol with 0.5% TritonX-100 v/v) for 10 min and washed three times with sterile water. Dried seeds were sown on 0.5 × Murashige and Skoog (MS) agar medium, stratified in dark for 2 days at 4°C and then cultivated in a climatic chamber (Percival) under 16 h light/8 h dark cycle, 21°C day and 19°C night temperature. For cultivation in soil, 2-week-old diploid or tetraploid seedlings were transplanted to the moist soil after ploidy measurements, then the pots were moved to an air-conditioned chamber with controlled long-day conditions (16 h light/8 h dark cycle, 21°C day and 19°C night temperature, 150 μmol photons m^–2^ s^–1^ light intensity provided by white-light tubes).

### Ploidy Measurements and Flow Cytometry

For tetraploid selection, plants grown from the big seeds produced by colchicine-treated plants were used. To determine the somatic ploidy levels, 1–2 young leaves were chopped with a razor blade in 500 μL Otto I solution (0.1M citric acid, 0.5% Tween 20 v/v). The nuclear suspension was filtered through 50 μm nylon mesh and stained with 1 mL of Otto II solution (0.4M Na_2_HPO_4_⋅12H_2_O) containing 2 μg DAPI (4′,6-diamidino-2-phenylindole). The ploidy was analyzed on a Partec PAS I flow cytometer with diploid WT plants used as an external standard. For the offspring ploidy measurements, seeds were collected per silique and all seeds per silique were sown (this avoids selection bias occurring when seed are collected per whole plant and the shrunk seeds are typically lighter and less round – thus often coming late during standard sowing procedures) and analyzed as described above.

### Hoyer’s Clearing

Flowers with green or white closed anthers were manually emasculated. Two days later, ovules were dissected and cleared by Hoyer’s solution as described (Liu and Meinke, 1998) with modifications. Dissolve 25 g Arabic gum in 25 mL distilled water in a glass beaker by heating to 60°C and stirring with a magnetic stirrer for about 1 h under a fume hood. Add 100 g chloral hydrate and keep dissolving until the solution will be clear and have an amber color. Subsequently add 10 mL glycerol, mix and keep the solution in dark at room temperature. Dissect ovules on a clear microscopic slide, add 20 μl Hoyer’s solution and mount with a 24 × 40 mm coverslip without applying a pressure. The slides were kept at 4°C overnight (or longer) and examined with an inverted microscope Olympus IX 83 using differential interference contrast (DIC) optics.

### Pollen Viability Assays

Fluorescein diacetate (FDA)-buffer mixture was prepared as described: 1 μL FDA (Sigma-Aldrich) stock solution (2 mg/mL in acetone) was added to 1 mL of BK buffer [0.127 mM Ca(NO3)2⋅4H2O, 0.081 mM MgSO4⋅7H2O, 0.1 mM KNO3, 15% Sucrose w/v and 10 mM MOPS, pH 7.5]. 20 μL FDA-buffer mixture was dripped to a microscopic slide then one opened flower was dropped into the FDA-buffer mixture and covered with 24 × 40 mm coverslip carefully. Data for pollen viability analysis were collected from at least three plants per genotype. The fluorescein fluorescence was observed after 20 min of staining using an inverted microscope Olympus IX 83: at 543/620 nm excitation/emission wavelengths and the same region was photographed with DIC optics to get the number of all pollen grains. ImageJ was used to merge the DIC and fluorescein channels.

### Cytological Experiments

The fixation of flower buds and chromosome spreads were carried out as described (Sánchez Moran et al., 2001), including minor modifications to adapt the protocol for the study of autopolyploids (Parra-Nunez et al., 2020). Data for cytological analyses were collected from at least three plants per genotype. Meiocytes were analyzed with an Olympus BX-61 epifluorescent microscope and images were captured with an Olympus DP-71 digital camera (Olympus, Germany). Using x100 magnification oil immersion objective resulted in an ultra-high image resolution of 4080 × 3072 and 46.40 pixels/μm. Manual mode was selected to allow the preferred image brightness to be set by clicking and dragging the slider positioned in the exposure time. The images were captured in grayscale and edited in Adobe Photoshop.

For mitotic chromosome number counting, fresh inflorescences were fixed in ethanol: chloroform: acetic acid (6:3:1) solution overnight at room temperature then enzymatically digested in 0.3% (w/v) cellulase Onozuka R-10 (Serva, Germany, catalog no. 1641903), cytohelicase from *Helix pomatia* (Sigma-Aldrich, St. Louis, catalog no. C8274) and pectolyase from *Aspergillus japonicus* (Sigma-Aldrich, St. Louis, catalog no. P3026) for 3 h at 37°C. After the enzymatic digestion, single flower buds were dissected and chopped in 60% acetic acid and slides were placed on the heating block for 2 min at 50°C. Then, cells on slides were fixed in Carnoy’s fixative. Chromosomes were counterstained with DAPI 1.5 μg/mL (Vector Laboratories, United States). All slides were examined with Axio Imager Z.2 Zeiss microscope (Zeiss, Germany) equipped with Cool Cube 1 camera (Metasystems, Germany). We used ×60 and ×100 objectives and filter for DAPI (emission spectrum ‘405 nm). Scale bars were adjusted to the objective that was applied. Image processing was carried out using ISIS software 5.4.7 (Metasystems, Germany) and Adobe Photoshop software (CS5).

### Software

Microsoft Office Excel 2016, PowerPoint 2016, GraphPad Prism 8.2.1, ImageJ 1.52p, Adobe Photoshop CS5 and Illustrator were used for graph and image composition.

## Results

### Tetraploidy Enhances Fertility Defects in *nse2-1* and *nse4a-2* Plants

**FIGURE 1:**
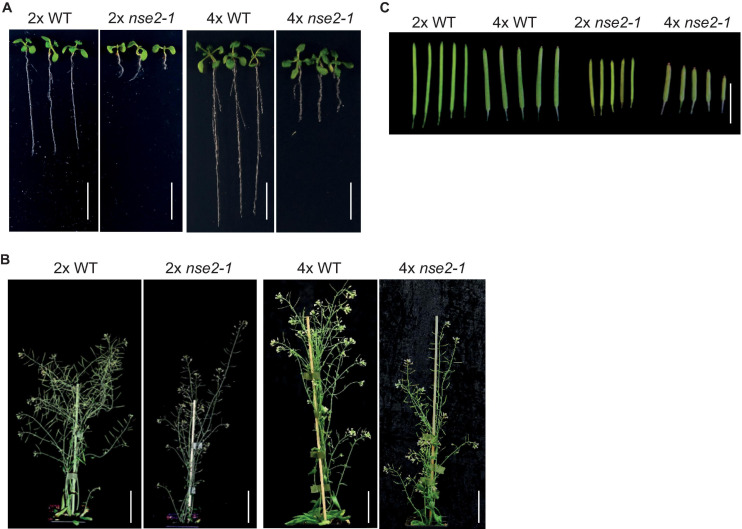
Developmental phenotype of diploid (2x) and tetraploid (4x) WT and *nse2-1* plants. **(A)** The phenotype of 2-weeks-old *in vitro* grown seedlings. Scale bar = 1 cm. **(B)** The whole plant phenotype of 5-week-old plants. Scale bars = 5 cm. **(C)** Representative siliques of the analyzed genotypes. Scale bar = 1 cm.

The diploid (2x) Arabidopsis *nse2* mutant plants have a significantly reduced fertility (Liu et al., 2014; Yang et al., 2021). To analyze the dosage-dependent role of *NSE2*, we produced autotetraploid wild-type (4x WT) and *nse2-1* (4x *nse2-1*) plants (Yang et al., 2021). With these lines, we noticed that 4x *nse2-1* plants had a reduced root length in the juvenile stage and a lower plant height at the adult stage compared with 4x WT (Figures 1A,B). The same differences were observed also between 2x *nse2-1* and 2x WT plants (Figures 1A,B), but both 4x WT and 4x *nse2-1* plants were bigger and had longer roots. In contrast, the siliques from 4x *nse2-1* were thicker but shorter than those of 2x *nse2*-1, possibly indicating that the autotetraploidy increases the seed size but enhances fertility defects of *nse2-1*, respectively (Figure 1C).

**FIGURE 2:**
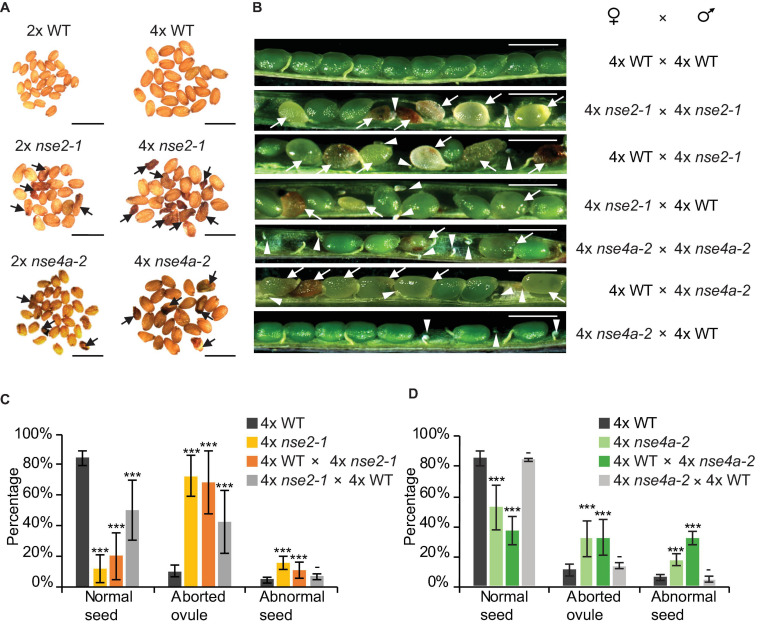
Reduced fertility of tetraploid *nse2-1* and *nse4a-2* plants. **(A)** Representative dry seeds of diploid (2x) and tetraploid (4x) WT, *nse2-1*, and *nse4a-2* plants. Arrows indicate examples of shrunken seeds. Scale bars = 1 mm. **(B)** Opened siliques 13 days after pollination (DAP). Aborted ovules are marked with arrowheads and abnormal seeds (typically shrunk, brown/pale, or partially transparent) with arrows. Scale bar = 1 mm. **(C,D)** Frequencies of normal seeds (NS), aborted ovules (AO) and abnormal seeds (AS) in manually pollinated 4x WT, 4x *nse2-1*, and 4x *nse4a-2* plants and their F1 reciprocal crosses. Error bars represent standard deviation among the means of three individual plants. Significance in Fisher’s exact test relative to 4x WT in the given group (NS, AO, AS): − = *P* > *0.05*, * = *P* < *0.05*, ** = *P* < *0.01*, and *** = *P* < *0.001*. Source values and basic counts are provided in Supplementary Table 1.

To further explore this observation, we analyzed the seed traits of 4x WT and mutant plants. We included also tetraploid *nse4a-2* (4x *nse4a-2*) because *NSE4A* is another subunit of SMC5/6 complex whose loss-of-function plants have fertility defects (Díaz et al., 2019). Dry seeds from 4x WT plants were larger than those from diploid WT (2x WT), but both were regular in shape and had a normal light brown color (Figure 2A). In contrast, both 2x and 4x mutants produced seeds with a variable shape, size, and color, including very large or little seeds, shrunk, and colored from normal to dark brown (Figure 2A). Analysis of siliques 13 days after self-pollination (DAP) revealed that 4x WT produced 84.3% normal seeds, 10.2% aborted ovules and 5.5% abnormal seeds (plants/siliques/seeds = 3/15/899, Figures 2B,C and Table 1). Equally old 4x *nse2-1* and 4x *nse4a-2* plants showed 11.6 and 51.8% normal seeds, 72.6 and 31.1% aborted ovules, and 15.8 and 17.1% abnormal seeds (plants/siliques/seeds = 3/15/739 and 3/15/843, respectively; Figures 2B–D and Table 1). Hence, the frequencies of both aborted ovules and abnormal seeds were significantly increased in 4x mutants compared to 4x WT (Fisher’s exact test, *P* < *0.00001*; Figures 2C,D and Supplementary Table 1). In addition, the comparison of the traits in 4x mutants relative to the 2x mutants (data from Yang et al., 2021) revealed that the ovule abortion and seed abnormality were statistically significantly more pronounced in the 4x *nse2-1* compared to the 2x mutant plants (Fisher’s exact test, *P* < *0.001*, Supplementary Table 2). This suggested ploidy-dependent fertility defects in tetraploid *nse2-1* mutants. However, no significant difference in the frequency of abnormal seeds was found for the 4x and 2x *nse4a-2* plants (Fisher’s exact test, *P* = 0.4299; Supplementary Table 2). It may be because *nse4a-2* is a partial loss-of-function allele (Díaz et al., 2019).

**TABLE 1:** Seed phenotype of self-pollinated and reciprocally crossed between tetraploid (4x) WT, *nse2-1*, and *nse4a-2* plants.

**Mother**	**Father**	**Events (n)**	**Trait (%)**		
			**Normal seeds**	**Aborted ovules**	**Abnormal seeds**
4x WT	4x WT	899	84.3	10.2	5.5
4x *nse2-1*	4x *nse2-1*	739	11.6	72.5	15.8
4x WT	4x *nse2-1*	691	20.3	68.7	11.0
4x *nse2-1*	4x WT	607	50.6	42.5	6.9
4x *nse4a-2*	4x *nse4a-2*	843	51.8	31.1	17.1
4x WT	4x *nse4a-2*	802	53.2	31.7	15.1
4x *nse4a-2*	4x WT	722	83.4	13.2	3.5

To test for the contribution of the parents to the abnormal seeds, we performed reciprocal crosses between 4x WT and 4x mutant plants. When 4x WT plants were fertilized by either 4x *nse2-1* or 4x *nse4a*-2, we observed 11.0 and 15.1% abnormal seeds 13 DAP (plants/siliques/seeds = 3/15/691 and 3/14/802, respectively; Figures 2B–D and Table 1). On contrary, when 4x WT was used to pollinate 4x *nse2-1* or 4x *nse4a-2*, there were only 6.9 and 3.5% abnormal seeds found (plants/siliques/seeds = 3/15/607 and 3/15/722, respectively). This generally matched 5.5% such seeds in self-pollinated 4x WT (plants/siliques/seeds = 3/15/899; Figures 2B–D and Table 1) and was significantly less than in the above mentioned reciprocal crosses (Fisher’s exact test, *P* < *0.05*; Supplementary Table 1). This suggests that the abnormal seed development is caused predominantly paternally, and to a minor extent also maternally, in 4x *nse2-1* and 4x *nse4a-2* mutants.

### Tetraploid *nse2* Leads to Defects in Both Male and Female Gametophytes

Previously, we showed that 2x *nse2-2* mutations cause ovule lethal defects (Yang et al., 2021). In contrast, the genetic material of the diploid microspores was at least partially transmissible and resulted in abnormal seed development. Here, we analyzed the female and male gametophyte development in the context of 4x *nse2-1* mutant plants.

**FIGURE 3:**
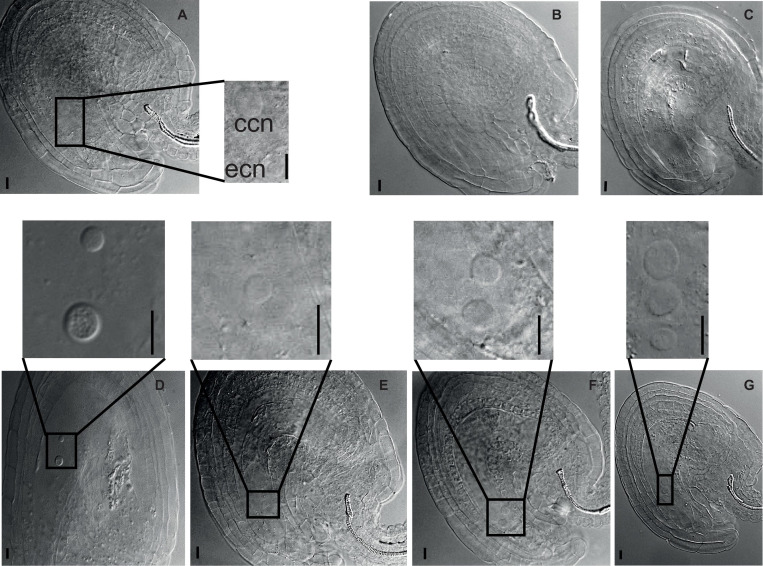
Developmental defects in female gametophyte of tetraploid *nse2-1*. Cleared ovules from 4x *nse2-1* were observed under a differential interference contrast microscope. The nuclei are marked with dashed circles and arrows. Scale bars = 10 μm. **(A)** A typical 4x WT-like embryo sac showing one egg cell nucleus (ecn) and one central cell nucleus (ccn). **(B–G)** 4x *nse2-1* ovules displaying specific defects: **(B)** absence of embryo sac and **(C)** embryo sacs **(C)** without nuclei, **(D)** with two nuclei at the abnormal position, **(E)** only one nucleus, **(F)** two equally size nuclei, and **(G)** one smaller nucleus and two bigger nuclei.

First, we inspected the female gametophyte development (Table 1). In crosses where 4x *nse2-1* was used as a mother, we found 42.5% aborted ovules. This suggests that 4x *nse2* has a pre-zygotic maternal dysfunction. To determine a possible source of this defect, we analyzed the morphology of 54 embryo sacs in 2x and 104 in 4x *nse2-1* plants, respectively. In 2x *nse2-1*, there were 13.0% (7 out of 54) WT-like embryo sacs, 38.9% (21 out of 54) ovules without embryo sacs, 22.2% (12 out of 54) embryo sacs without nucleus and 25.9% embryo sacs with three nuclei (14 out of 54). In 4x *nse2-1*, only 4.8% of ovules (5 out of 104) carried WT-like embryo sacs (with a smaller egg cell nucleus and larger central cell nucleus positioned closer and more distant to the micropylar pole, respectively) and the majority of the ovules (95.2%; 99 out of 104) showed diverse defects (Figure 3). In total, 72.1% (75 out of 104) ovules fully lacked an embryo sac or it was without detectable nuclei (Figures 3B,C). In 23.1% (24 out of 104) of the ovules, embryo sacs contained nuclei, but they deviated from the WT parameters. There was only one nucleus, three nuclei, or occasionally also two nuclei that were abnormally positioned (Figures 3D–G). This suggests that tetraploidy in combination with *nse2-1* mutation results in severe female pre-zygotic sterility, but at least some of the embryo sacs with abnormal nuclei can be fertilized.

**FIGURE 4:**
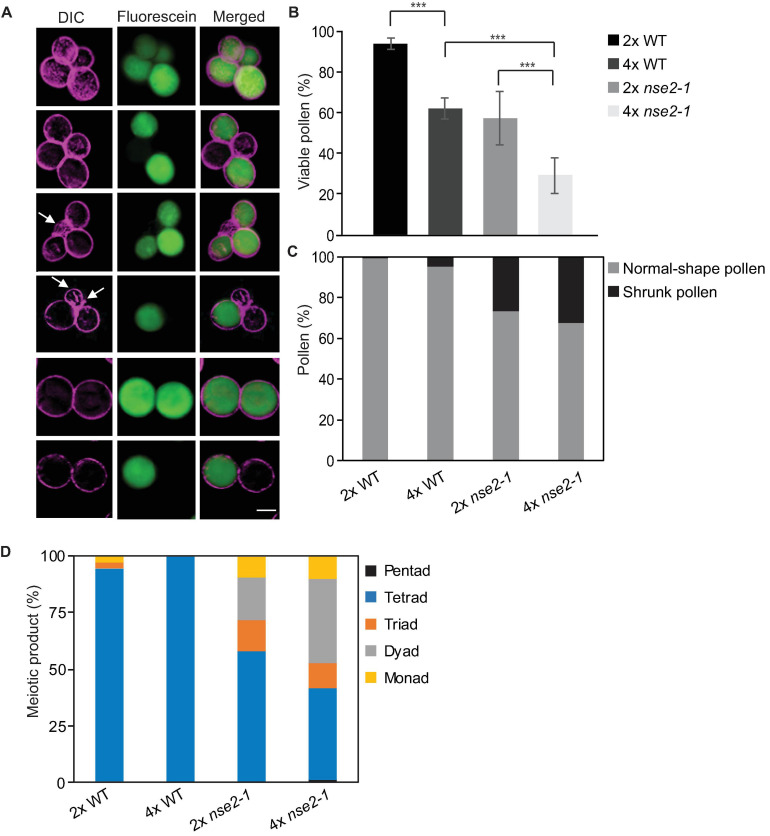
Microspore phenotype of tetraploid (4x) *nse2-1* plants. **(A)** Representative mature pollen stained by fluorescein diacetate from 4x *nse2-1 qrt1-4* plants. Differential interference contrast (DIC) images were pseudocolored in violet. Viable microspores are indicated by fluorescein signals (green). Shrunk microspores are indicated by arrows. Scale bar = 20 μm. **(B)** Frequencies of viable pollen from 2x WT, 4x WT, 2x *nse2-1*, and 4x *nse2-1* (all in *qrt1-4* background). Error bars represent standard deviations among the means of two or three individual plants. Significance in Fisher’s exact test: − = *P* > *0.05*, * = *P* < *0.05*, ** = *P* < *0.01*, and *** = *P* < *0.001*. Source values and basic counts are provided in Supplementary Table 3. **(C)** Quantification of normal-shape and shrunken pollen observed in 2x WT, 4x WT, 2x *nse2-1*, and 4x *nse2-1* in *qrt1-4* background. **(D)** Quantification of meiotic products with different numbers of microspores as observed in 2x WT, 4x WT, 2x *nse2-1*, and 4x *nse2-1* in *qrt1-4* background.

Second, we assessed several male gametophyte traits. Using fluorescein diacetate (FDA) assay, we quantified the microspore viability. This revealed 29.4% (375 out of 1275) viable pollen in 4x *nse2-1* which was significantly less than 62.2% (941 out of 1512, Fisher’s exact test, *P* < *0.00001*) of such pollen in 4x WT plants (Figures 4A,B). It has to be noted that both 4x WT and *nse2-1* had also significantly less viable pollen compared to 2x WT and 2x *nse2-1* (95.3 and 65.0%, respectively; based on published data of Yang et al. (2021); Fisher’s exact test, *P* < *0.00001*) grown under the same cultivation conditions (Supplementary Table 3).

The 4x WT and 4x *nse2-1* genotypes used in this study were produced in the *qrt1-4* mutant background and were representing 4x WT *qrt1-4* single and 4x *nse2-1 qrt1-4* double mutants. The *qrt1* mutations cause a stable association of the microspores arising from one meiosis which allows scoring for a constitution of the male meiotic products and also for abnormally developed (small and shrunk) microspores (Preuss et al., 1994). In 4x *nse2-1 qrt1-4*, we found 32.5% (414 out of 1275) shrunk microspores (Figure 4A, arrows), which is similar with the 27% in 2x *nse2-1 qrt1-4* plants (Figure 4C). However, both 4x and 2x WT *qrt1-4* plants showed much lower frequencies (5.0 and 0.7%, Figure 4C) of shrunken microspores in our experiments (75 out of 1512 and 4 out of 575, respectively; Figure 4A, arrows; Supplementary Table 4). This suggests that 4x WT plants have a seven-fold higher frequency of pollen abortion compared to the 2x WT and the pollen abortion rate remained similarly high in the mutants irrespective of their ploidy. Finally, we scored how many microspores were produced from one meiotic division (irrespective of their viability and shape). All meiotic products were tetrads in 4x WT *qrt1-4*, indicating that these plants undergo normal reductional division. On contrary, 4x *nse2-1* plants produced less than half (40.4%) of microspores in tetrads (Figure 4D). The remaining meiotic products were monads (9.6%), dyads (37.8%), triads (11.2%), and rarely even pentads (1.1%). This suggests abnormal meiosis in 4x *nse2* with the possible absence of the reductional divisions (monads to triads) or multipolar spindle (pentads).

Taken together, our results showed that 4x *nse2* plants produce a high number of abnormal male and female gametes.

### Defective Male Meiosis Leads to Unreduced and Aneuploid Microspores in 4x *nse2-1* Plants

Meiotic progression in 2x *nse2* pollen mother cells takes place normally at prophase I. During metaphase I five bivalents are formed, but they are more stretched and elongated than in 2x WT. At anaphase I, chromosome fragments are present in most cells. During second meiotic division different problems are revealed such as non-reduced nuclei, extensive chromosome fragmentation, and chromosome bridges, among others (Yang et al., 2021).

**FIGURE 5:**
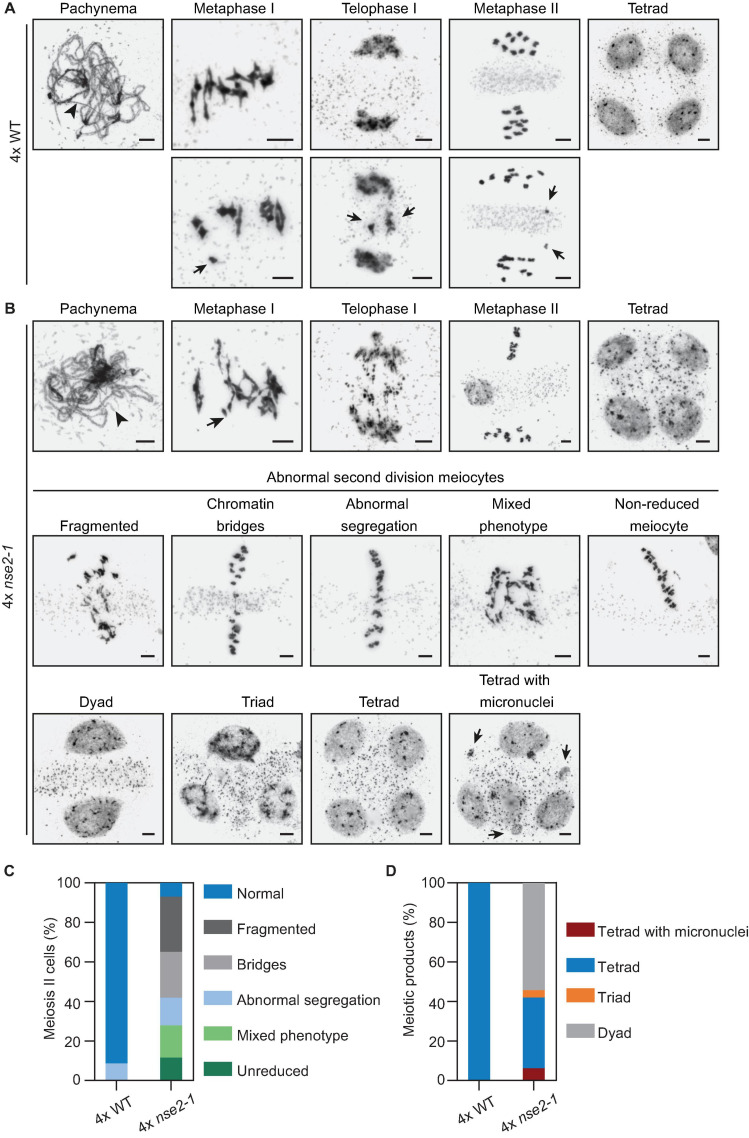
Analysis of male meiosis in 4x *nse2-1* plants. **(A)** Representative images of selected meiotic stages in 4x WT plants. Top row: Unsynapsed regions are detected in pachynema (arrowhead). At metaphase I, the chromosome associations are mostly bivalents and quadrivalents. In most meiocytes, chromosome segregation is correct, both in the first and second meiotic division, resulting in the formation of tetrads with balanced nuclei. Bottom row: Examples of metaphase I with a univalent, telophase I with a delay in chromosome segregation, and a metaphase II with chromatids resulting from the equational segregation of anaphase I univalent. **(B)** Representative images of the phenotype observed in 4x *nse2-1* plants. As well as in WT plants, there were some unsynapsed regions at pachynema (arrowhead). At metaphase I, an increase in the frequency of univalents (arrow) was detected with respect to the WT. Complex entanglements were also frequent at this stage. Telophases I displayed a high frequency of chromosome fragments. Meiotic problems were also evident during the second meiotic division in almost all cells analyzed, including chromosome fragmentation, chromatin bridges and/or abnormal chromosome segregation, and non-reduced meiocytes. At the end of the meiotic division, dyads, triads, tetrads, and tetrads with micronuclei were formed (bottom row). See the text for more details. Scale bars = 5 μm. **(C)** Quantification of the different phenotype observed in 4x *nse2-1* plants during second meiotic division. **(D)** Quantification of division products at the end of meiosis. Note: The data for 2x WT and 2x *nse2* plants were published previously (Yang et al., 2021; Figures 3, 4).

Similar to the situation in 2x *nse2*, we found no apparent differences between 4x *nse2-1* and 4x WT plants at prophase I. In both genetic backgrounds, we observed nearly complete synapsis at pachynema with some unsynapsed regions due to the presence of synaptic partner switches produced by multivalent associations involving three or even four chromosomes (Figures 5A,B, arrowheads). At metaphase I, the different multivalent associations in tetraploids depend on the pattern of CO formation among the four homologous chromosomes. In 4x WT plants, we observed bivalents and quadrivalents, but occasionally also trivalents and univalents (Figure 5A, arrowheads). In 4x *nse2-1* plants, we did not detect apparent differences in chromosome associations from WT, with bivalent and quadrivalent associations also being the majority (Figure 5B). Nevertheless, the frequency of cells with univalents was twice (14.81%, 4 out of 27) that of the WT (7.30%, 6 out of 82). Chromatin did not appear normal either, due to the frequent presence of constrictions and even fragments, which was similar to the observations in 2x *nse2* plants (Yang et al., 2021). During anaphase I and telophase I, chromosomal fragmentation increased, spanning the region between the segregating chromosomes, being evidenced in all 45 cells analyzed (Figure 5B). During these stages, we did not observe fragmentation in 4x WT plants in any case and we only detected chromosome laggards in one of the cells analyzed (6.25%, 1 out of 16) (Figure 5A, bottom row, arrows).

In the second meiotic division, the defects in 4x *nse2-1* plants were more drastic than in the first meiotic division, as a result of an accumulation of errors (Figures 5B,C). Meiotic irregularities were detected in almost all analyzed cells (93.0%, 40 out of 43), namely: (i) chromosome fragmentation (27.9%), (ii) chromatin bridges (23.3%), (iii) abnormal segregation (13.9%), (iv) meiocytes with several problems including chromosomal bridges and fragments (16.3%), and (v) non-reduced meiocytes (11.6%). In contrary, only 68.75% of the meiocytes have second meiotic division defect in 2x *nse2* pollen mother cells (Yang et al., 2021). In 4x WT plants, the irregularities were also detected in a low percentage of meiocytes (8.6%, 13 out of 150) during the second meiotic division (Figure 5C). On contrary to 4x *nse2-1*, chromosomal bridges or fragments were never observed in 4x WT plants. However, they displayed incorrect segregation of one or two chromosomes (possibly due to the formation of univalents and multivalents in metaphase I).

At the end of the meiotic division, only tetrads (*n* = 134) with apparently balanced nuclei were observed in 4x WT plants (Figures 5A,D), although we cannot exclude occasional aneuploidies (as a consequence of improper segregation during the second meiotic division). These aneuploidies would only affect one or two chromosomes. In 4x *nse2-1* plants, we observed dyads (54.3%, 44 out of 81), triads (3.7%, 3 out of 81), tetrads (35.8%, 29 out of 81), and even tetrads with micronuclei (6.17%, 5 out of 81) (Figures 5B,D). In 2x *nse2* plants the tetrads represented 49.2% and no micronuclei were observed (Yang et al., 2021). In summary, analysis of meiosis in 4x *nse2-1* plants revealed that the defects during later stages of meiosis were enhanced compared to 2x *nse2-1* plants.

### Tetraploid *nse2-1* and *nse4a-2* Plants Produce Aneuploid Offspring

The spectrum of meiotic defects in 4x *nse2-1* plants prompted us to analyze the ploidy levels of their progeny by flow cytometry. Among the offspring from self-pollinated 4x WT plants (*n* = 120), we found 96.7% tetraploids (117 out of 120) and 3.3% (3 out of 120) putative aneuploids (Table 2). The three putative aneuploid WT plants showed minimal shifts of the flow cytometry peaks relative to the known tetraploid control. This is in contrast with an earlier study which showed that natural and synthetic 4x WT Arabidopsis plants produced about 30% aneuploid progeny (Henry et al., 2006). Since Henry and colleagues used more sensitive detection methods and also pointed to a lower sensitivity of flow cytometry to detect single chromosome addition/loss genotypes, our frequencies are most likely an underestimation.

**TABLE 2:** Flow cytometry-based ploidy levels of F1 offspring plants from tetraploid (4x) WT, *nse2-1*, and *nse4a-2* parents.

**Genotype**		**Germination rate (%)**	**Events (n)**	**Ploidy (%)**				
**Mother**	**Father**			**Euploid**		**Aneuploid**		
				**4x**	**6x**	**Total**	**+**	**−**
4x WT	4x WT	72.7%	120	96.7	0.0	3.3	100.0	0.0
4x *nse2-1*	4x *nse2-1*	48.7%	92	32.6	19.6	47.8	70.5	29.5
4x WT	4x *nse2-1*	66.3%	163	75.4	8.6	16.0	88.5	11.5
4x *nse2-1*	4x WT	84.9%	247	84.2	1.2	14.6	69.4	30.6
4x *nse4a-2*	4x *nse4a-2*	89.1%	196	85.2	6.6	8.2	68.8	31.2
4x WT	4x *nse4a-2*	55.8%	91	91.2	4.4	4.4	100.0	0.0
4x *nse4a-2*	4x WT	88.6%	124	93.5	0.0	6.5	75.0	25.0

*4x = tetraploid, 6x = hexaploid. + = DNA gain, − = DNA loss.*

**FIGURE 6:**
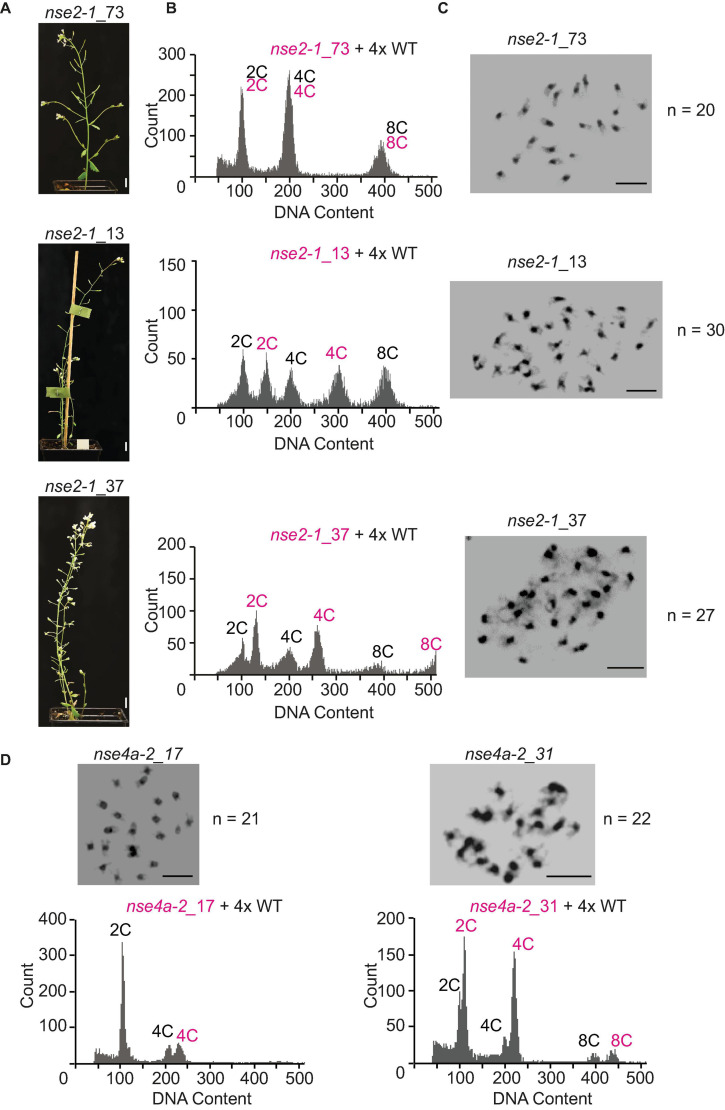
Flow-cytometry ploidy determination in offspring of 4x *nse2-1* and 4x *nse4a-2* plants. **(A)** Phenotype of selected 4-weeks-old plants of *nse2-1*_73, *nse2-1*_13, and *nse2-1*_37. Scale bars = 1 cm. **(B)** Flow-cytometry histograms of plants shown in panel **(A)**, indicating that *nse2-1*_73 is tetraploid, *nse2-1*_13 hexaploid, and *nse2-1*_37 aneuploid. For the ploidy measurements, the nuclei suspension was prepared by mixing leaves from tested *nse2-1* plants and 4x WT control. Multiple peaks correspond to nuclei of different C content as indicated. **(C)** Mitotic figures of *nse2-1*_73, *nse2-1_13*, and *nse2-1*_37 plants. The number of chromosomes is given next to the figures. Scale bars = 5 μm. **(D)** Mitotic metaphase plates and flow-cytometry histograms of the ploidy level of two selected aneuploid *nse4-2* plants. Chromosome numbers are given right of the figures. Scale bars = 5 μm. The ploidy levels were measured by preparing the nuclei suspension from a mix of the leaves the *nse4a-2* candidate plant and 4x WT control. Note that an addition of a single or two chromosomes is clearer visible at 4C peaks (or 8C peaks if visible).

Among 92 plants derived from self-pollinated 4x *nse2-1*, we found 32.6% tetraploids (30 out of 92), 47.8% putative aneuploids (44 out of 92), and 19.6% hexaploids (18 out of 92) (Table 2 and Figure 6). The hexaploids were most likely a product of a fusion of one reduced and one unreduced gamete. The flow cytometry analysis of the aneuploid mutant plants indicated that 70.5% (31 out of 44) gained and 29.5% (13 out of 44) lost one or more chromosomes (Table 2). In the self-pollinated 4x *nse4a-2* (*n* = 196), we found 85.2% tetraploids (167 out of 196), 8.2% putative aneuploids (16 out of 196), and 6.6% hexaploids (13 out of 196) (Table 2). Similar to 4x *nse2-1*, about two-thirds (68.7%) of the putative aneuploids gained and one-third (31.3%) lost one or more chromosomes (Table 2).

To confirm the aneuploidy, we performed cytology analysis on the selected candidates that appeared to have either extra or missing chromosomes (Figure 5C). To address the parental contribution to the ploidy changes in the progeny, we performed reciprocal crosses between 4x WT and 4x *nse2* plants and analyzed the ploidy of the resulting plants (Table 2). The hexaploidy was caused 7.2-fold more frequently by the unreduced paternal over the maternal 4x *nse2-1* gametes (8.6 versus 1.2%, respectively; Table 2). In contrast, the aneuploidy was caused equally by the paternal (15.3%) and the maternal (14.2%) 4x *nse2-1* gametes (Table 2). We found similar trends for the induction of hexaploidy and aneuploidy in the progeny of 4x *nse4a-2*. There were 4.0% hexaploids in 4x WT × 4x *nse4a-2* crosses versus 0% hexaploids in reciprocal crossing direction (Table 2). The aneuploid offspring arose almost equally from both paternal and maternal gametes (4.4 and 6.5%, respectively; Table 2).

Hence, the 4x SMC5/6 complex mutants produce higher-polyploid and also aneuploid offspring from both parents.

## Discussion

Here, we analyzed the effects of polyploidy on the genome stability and reproductive success in the background of autotetraploid Arabidopsis SMC5/6 complex deficient mutants. Most of our experiments focused on *NSE2*, which encodes an important, but in Arabidopsis non-essential, E3 SUMO ligase subunit of the SMC5/6 complex (Ishida et al., 2009). For a subset of experiments, we analyzed also *NSE4A*, as the kleisin subunit of the SMC5/6 complex that is active in both somatic and reproductive tissues and is essential for plant survival (Díaz et al., 2019). The strong loss-of-function alleles of *NSE4A* are lethal in Arabidopsis and the *nse4a-2* allele used here is a partial loss-of-function mutant. This is in agreement with our observations that the defects of both mutants are similar, but those of *nse4a-2* plants are generally weaker.

The SMC5/6 complex has multiple functions during meiosis. It is required for the repair of SPO11-induced DNA double-strand breaks and its absence produces a severe chromosome fragmentation due to the presence of entanglements and concatenations generated as a consequence of an accumulation of joint molecules (JM) (Copsey et al., 2013; Xaver et al., 2013; Menolfi et al., 2015). A recent study from Arabidopsis also suggested that RAD51 restrains the SMC5/6 complex from inhibiting the activity of meiotic recombinase DMC1 (Chen et al., 2021). In addition, *nse2* mutants generate diplogametes (Yang et al., 2021). The non-reduced nuclei result from cells with an abnormal spindle organization in which organelles are not organized in a defined band after telophase I. Interestingly, this trait is recombination-independent. In this study, we analyzed the consequences of polyploidy in the SMC5/6 complex mutants to find whether the duplication of the entire genome will buffer or enhance the defects present in the diploid mutant and whether there will be new features compared to WT plants. We observed a higher frequency of univalents in 4x *nse2* compared to the 4x WT plants. However, in the diploid mutant, five bivalents are invariably formed, as in the diploid control (Yang et al., 2021). The defects of the tetraploid mutant were more drastic in the second meiotic division. A higher percentage of meiocytes with abnormalities was observed with respect to the diploid mutant (93 *vs.* 69%, see also Yang et al., 2021). This could be explained by the role of the SMC5/6 complex in the HR process (Chen et al., 2021), which may be more important in the autotetraploid context as suggested by our data. The accumulation of JMs would be higher in a situation in which the chances of finding homologous sequences to recombine are increased (Voorrips and Maliepaard, 2012). On the other hand, in 4x *nse2* plants we detected a lower percentage (nearly half) of second division meiocytes displaying all chromosomes on one side of the organelle band (non-reduced) compared to 2x *nse2* plants (11.63 *vs.* 19.40%, see also Yang et al., 2021). Although the percentage of dyads was similar in the tetraploid and the diploid *nse2-1* plants, a lower percentage of tetrads was detected in 4x *nse2* plants. In the 4x mutant, we also observed tetrads with micronuclei, which were not formed in the case of the 2x mutant. As mentioned above, non-reduced meiocytes appear as a consequence of recombination-independent problems generated by the absence of the SMC5/6 complex. In our recent study focusing on the meiosis of 2x *nse2* plants, we showed that this may be related to the organization of the spindle, to the interaction of the kinetochores and the spindle or to delays in chromosome segregation (Yang et al., 2021). In this context, the improper localization of a defined organelle band prevents the formation of two defined nuclei with five chromosomes each (2x *nse2*) or ten chromosomes each (4x *nse2*). The fact that the frequency of non-reduced meiocytes is lower in the 4x relative to the 2x mutant can be explained by the increase in the number of chromosomes. The location of all twenty chromosomes in a single nucleus is less likely than the location of the ten chromosomes. This would also explain why there are more aneuploidies in the tetraploid mutant and a greater reduction in fertility.

The meiotic irregularities in SMC5/6 complex mutants have also profound effects on the seed development and offspring genomic constitution. The 4x *nse2* plants are almost sterile, with less than 10% normal seeds, compared to about 35% such seeds in the 2x mutant. This is due to a strongly affected ovule development leaving only about 30% of ovules capable of seed development in 4x *nse2-1*. Many of the developing seeds are aneuploid, represented mostly by addition of one or two chromosomes. Importantly, the aneuploidy was caused equally from both maternal and paternal sides. Rarely, also unreduced female gametes of tetraploid *nse2* plants gave rise to the hexaploid offspring. These are tetraploidy-associated characters because we observed neither the aneuploidy offspring nor the viable unreduced female gametes in 2x *nse2* plants.

The analysis of polyploids makes it possible to explore some aspects of meiosis more in depth compared to diploids, since in a polyploid condition the chances of pairing and finding homologous sequences to recombine increase. Altogether, our results highlight that the mutations in the SMC5/6 complex cause partially common, but also some unique characters when comparing the phenotypes of diploid and the tetraploid plants. A similar situation has been described in other mutants, for example, those affecting suppressors of recombination like *FANCONI ANEMIA COMPLEMENTATION GROUP M* (*FANCM*). The *fancm* mutants produce a significant increase in HR in diploid plants (Crismani et al., 2012; Li et al., 2021). However, silencing FANCM in tetraploid plants has less or no effect on recombination (Blary et al., 2018; Raz et al., 2021). This indicates that due to the specificities of tetraploid meiosis, the importance of certain molecular factors and complexes may increase or decrease. In summary, the described defects highlight the importance of studying the consequences of mutations in genes affecting meiosis and reproductive development in diploid versus polyploid conditions, especially in the crop species, where polyploids could provide the potential to increase agriculturally important traits.

## Data Availability Statement

The original contributions presented in the study are included in the article/Supplementary Material, further inquiries can be directed to the corresponding author.

## Author Contributions

AP, FY, and MP designed the project. FY performed plant phenotypic characterization, crosses, analysis of pollen, and ploidy measurements. NF prepared and analyzed meiocytes. JM counted mitotic chromosome numbers. AP and FY wrote the manuscript with help of other authors. All authors approved the submitted version.

## Conflict of Interest

The authors declare that the research was conducted in the absence of any commercial or financial relationships that could be construed as a potential conflict of interest.

## Publisher’s Note

All claims expressed in this article are solely those of the authors and do not necessarily represent those of their affiliated organizations, or those of the publisher, the editors and the reviewers. Any product that may be evaluated in this article, or claim that may be made by its manufacturer, is not guaranteed or endorsed by the publisher.

## Footnotes

1
http://signal.salk.edu/cgi-bin/tdnaexpress
