# Supplementary material for: Structural Maintenance of Chromosomes 5/6 Complex Is Necessary for Tetraploid Genome Stability in Arabidopsis thaliana
Source: Front Plant Sci. 2021 Oct 5;12:748252. doi: 10.3389/fpls.2021.748252 (PMC8525318; doi:10.3389/fpls.2021.748252)
Supplement: Supplementary file 1 [file Table_1.DOCX]

**Supplementary materials**

SMC5/6 complex is necessary for tetraploid genome stability in *Arabidopsis thaliana*

Fen Yang^1,2^, Nadia Fernández Jiménez^3^, Joanna Majka^1,4^, Mónica Pradillo^3^, Ales Pecinka^1*^

^1^Institute of Experimental Botany, Czech Acad Sci, Centre of the Region Haná for Biotechnological and Agricultural Research, Šlechtitelů 31, 77900 Olomouc, Czech Republic

^2^Department of Cell Biology and Genetics, the Faculty of Natural Sciences, Palacký University, Šlechtitelů 27, 77900, Olomouc, Czech Republic

^3^Department of Genetics, Physiology and Microbiology, Faculty of Biology, Universidad Complutense de Madrid, 28040 Madrid, Spain

^4^Institute of Plant Genetics, Polish Academy of Sciences, Strzeszyńska 34, 60-479 Poznań, Poland
